# Mapping physiological inputs from multiple photoreceptor systems to dopaminergic amacrine cells in the mouse retina

**DOI:** 10.1038/s41598-017-08172-x

**Published:** 2017-08-11

**Authors:** Xiwu Zhao, Kwoon Y. Wong, Dao-Qi Zhang

**Affiliations:** 1 Eye Research Institute, Oakland University, Rochester, MI United States; 2 Department of Ophthalmology and Visual Sciences, University of Michigan, Ann Arbor, MI United States; 3 Department of Molecular, Cellular & Developmental Biology, University of Michigan, Ann Arbor, MI United States

## Abstract

In the vertebrate retina, dopamine is synthesized and released by a specialized type of amacrine cell, the dopaminergic amacrine cell (DAC). DAC activity is stimulated by rods, cones, and melanopsin-expressing intrinsically photosensitive retinal ganglion cells upon illumination. However, the relative contributions of these three photoreceptor systems to the DAC light-induced response are unknown. Here we found that rods excite dark-adapted DACs across a wide range of stimulation intensities, primarily through connexin-36-dependent rod pathways. Similar rod-driven responses were observed in both ventral and dorsal DACs. We further found that in the dorsal retina, M-cones and melanopsin contribute to dark-adapted DAC responses with a similar threshold intensity. In the ventral retina, however, the threshold intensity for M-cone-driven responses was two log units greater than that observed in dorsal DACs, and melanopsin-driven responses were almost undetectable. We also examined the DAC response to prolonged adapting light and found such responses to be mediated by rods under dim lighting conditions, rods/M-cones/melanopsin under intermediate lighting conditions, and cones and melanopsin under bright lighting conditions. Our results elucidate the relative contributions of the three photoreceptor systems to DACs under different lighting conditions, furthering our understanding of the role these cells play in the visual system.

## Introduction

In the vertebrate retina, a subpopulation of amacrine cells are dopamine-releasing neurons. These cells are referred to as dopaminergic amacrine cells (DACs). DACs are located in the inner nuclear layer (INL) of the retina, with a dense plexus of dendrites and axon-like processes in the inner plexiform layer (IPL) and clusters of axon-like processes in the outer plexiform layer (OPL)^1^. Through compartmental (synaptic) and volume (extrasynaptic) transmission, dopamine released from DACs influences virtually all levels of retinal circuitry and all major classes of retinal neurons. For instance, dopamine can restructure retinal function according to the prevailing illumination by modulating electrical and chemical synapses between retinal neurons and by altering the intrinsic properties of retinal neurons^2–8^.

Our previous work has suggested that DACs are excited by synaptic input from light-increment (ON) cone bipolar cells upon illumination^9^. This work has been supported by several anatomical studies demonstrating that ON cone bipolar cells contact DACs through conventional ribbon synapses in the inner IPL^10^ and ectopic ribbon synapses in the outer IPL^11, 12^. ON cone bipolar cells are not only driven directly by cone photoreceptors, but also directly and indirectly by rods^13, 14^. However, rod- and middle-wavelength-sensitive cone (M-cone)-mediated DAC responses are not easily distinguishable in the wild-type retina due to the overlapping spectral sensitivities of rods (λ_max_ = 500 nm), M-cones (λ_max_ = 508 nm) and melanopsin (λ_max_ = 479 nm)^15, 16^. Using a genetic mouse model in which cones are the only light-sensitive cells, we have extensively examined cone-mediated DAC responses^17^. However, as those studies were performed in partially light-adapted retinae, the sensitivity of cone-mediated DAC responses in dark-adapted retinae is not known.

In addition, rod-mediated DAC responses have not been examined in the rod-pathway isolated system. One previous report utilized wild-type retinae with a focus on studying rod-driven inhibitory responses (but not excitatory responses) in DACs under dim light^18^. In this study, rod-mediated excitatory responses (if any) at high light intensities were not separated from M-cone- or melanopsin-mediated responses in DACs. Given that our knowledge of rod function has been expanded by recent studies demonstrating rod involvement in cone-like spatial visual functions, circadian photoentrainment, and visually-driven ocular growth^19–22^, it would be of interest to determine whether rods excite DACs under bright as well as dim light intensities. Furthermore, Newkirk *et al*. demonstrated that DACs adapt to steady and flickering background lights^18^, two stimuli that have been previously reported to increase dopamine release in cold-blooded vertebrate and mammalian retinae^4, 23–26^. However, it remains unknown how rods and M-cones contribute to the response of DACs to an adapting light under dim and bright light conditions.

A growing body of evidence has demonstrated that DACs receive retrograde input from a third class of photoreceptor, the melanopsin-expressing intrinsically photosensitive retinal ganglion cell (ipRGC)^18, 27–31^. The melanopsin-based DAC response has been characterized as having a long latency, marked post-stimulus persistence, and a peak spectral sensitivity of 478 nm^27^. However, these previous studies were performed in partially light-adapted retinae. The sensitivity and kinetics of the melanopsin-based DAC response have not been examined in a fully dark-adapted retina. To fully characterize the relative contributions of rods, M-cones, and melanopsin to DACs, it is essential to compare the sensitivity and kinetics of the various photoreceptor inputs in the dark-adapted retina as well as the light-adapted retina.

The relative contributions of rods, M-cones, and melanopsin to DACs are affected by the distribution of opsins throughout the retina and the total number of each type of photoreceptor. In the mouse retina, rods make up approximately 97% of the classical photoreceptors, with an even distribution of rhodopsin throughout the retina^32, 33^. The remaining outer retinal photoreceptors are cones, which can be further classified by their peak sensitivity^32, 33^. About 5% are short (S)-wavelength cones, while 95% are short/middle (S/M)-wavelength cones^34, 35^. S/M cones co-express S-opsin and M-opsin in a dorsal-ventral gradient: S-opsin is predominantly expressed in the ventral retina, while M-opsin is dominant in the dorsal retina^35–38^. Although ipRGCs are classified into five subtypes (M1-M5)^39^, it is likely that M1 ipRGCs, which make up approximately 1% of all RGCs, drive DACs^30^. The density of M1 ipRGCs is slightly higher in the dorsal retina than in the ventral retina^40^.

In the present study, we utilized a combination of genetic, pharmacological, and multi-photon imaging approaches to examine the excitatory responses of DACs to light pulses across a wide range of intensities in the dark-adapted mouse retina. Our results suggest that rods contribute equally to DAC responses in the dorsal and ventral retina, but that M-cones and melanopsin contribute to dorsal DAC responses much more than to ventral DAC responses. We also examined the response of DACs to adapting light stimuli and found that DAC responses are mediated by rods under dim light conditions, rods/M-cones/melanopsin under intermediate light conditions, and M-cones/melanopsin under bright light conditions.

## Results

Several lines of transgenic mice in which DACs are labeled by red fluorescent protein (RFP) under the control of the *tyrosine hydroxylase* (*TH*) promoter were used for the present study. These were cone photoreceptor-specific cyclic nucleotide channel *Cnga3* ^−/−^ *TH*::RFP (referred to as “cone-function-knockout” mice), photopigment melanopsin *Opn4* ^−/−^ *Cnga3* ^−/−^ *TH::*RFP (“rod-function-only”), rod-specific G protein transducin α-subunit *Gnat1* ^−/−^ *Opn4* ^−/−^ *TH::*RFP (“cone-function-only”), *Gnat1* ^+/+^ *Cnga3* ^+/+^ *Opn4* ^+/+^ *TH::*RFP (“wild-type”), and connexin 36 (Cx36) knockout *TH*::RFP (“Cx36 KO”) mouse lines. We visualized RFP-labeled DACs in a flat-mount retina using a 915-nm multiphoton laser, with a <10 second laser exposure time per retina. This protocol has previously been shown to fully preserve the dark-adapted state of the retina^41^. Whole-cell recordings were made from RFP-labelled DACs voltage-clamped at *E*_*Cl*_ (−70 mV) to detect light-evoked excitatory postsynaptic currents (EPSCs). In the first set of experiments, we measured the response of DACs in dark-adapted retinae to a series of increasingly intense light stimuli. The second set of experiments explored pathways that could convey rod signals to DACs. In the last set of experiments, we tested the response of DACs to an adapting light (steady background illumination or low-frequency flashing light) at dim, intermediate and bright intensity levels.

### Intensity-response relations of dorsal and ventral DACs in dark-adapted wild-type retinae

**Figure 1:**
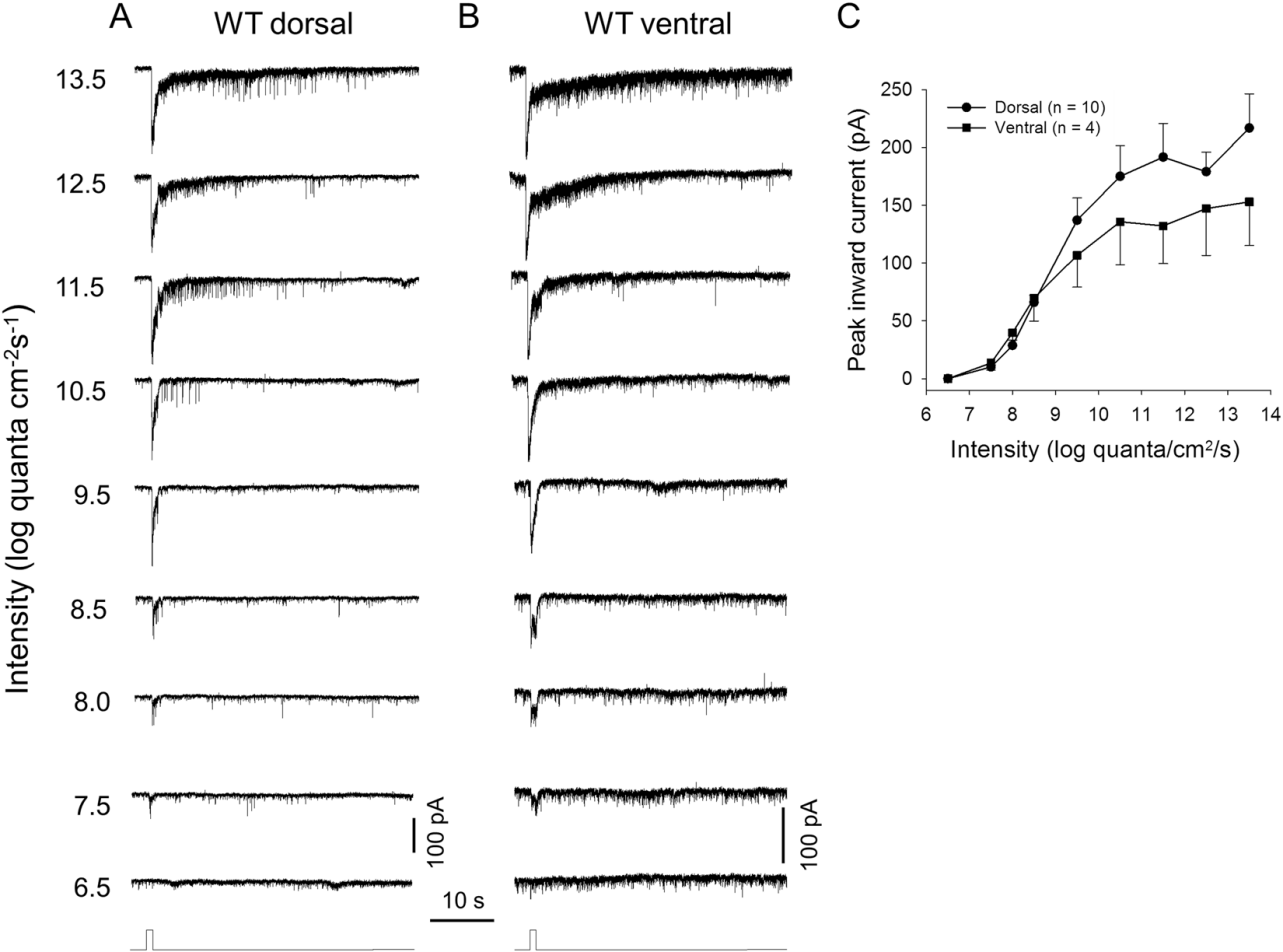
Light-evoked excitatory postsynaptic currents (EPSCs) of dorsal and ventral DACs in dark-adapted wild-type retinae. Light-evoked EPSCs of a dorsal DAC (**A**) and a ventral DAC (**B**) in response to a series of increasingly bright white light pulses (1-s duration) ranging from 6.5 to 13.5 log quanta/cm^2^/s. Stimulation bar shows timing of light pulse. (**C**) Intensity-response profiles showing the average peak amplitudes of DAC light-evoked EPSCs as a function of the light intensity. Closed circles: dorsal DACs; closed squares, ventral DACs. Each data point shows the average and standard error (SE) of multiple cells.

As we described above, S-opsin is predominantly expressed in the ventral retina, while M-opsin and melanopsin are dominant in the dorsal retina^35–38^. It is thus hypothesized that M-cones and melanopsin contribute differentially to dorsal and ventral DACs. To test this hypothesis, we measured DAC responses in the dorsal and ventral retina separately and compared them. We first examined the response of DACs in dark-adapted wild-type (WT) retinae to 1-s or 3-s white light stimuli ranging in intensity from 6.5 to 13.5 log quanta/cm^2^/s. In the dorsal retina, we found that a light pulse of 7.5 log quanta/cm^2^/s (near the rod threshold) began to evoke a small but obvious EPSC at light onset (Fig. 1A). The peak amplitude of the inward current gradually increased as the light intensity increased and reached 216 ± 94 pA (n = 10) at 13.5 log quanta/cm^2^/s (Fig. 1A,C). When the stimulus intensity was 9.5 log quanta/cm^2^/s or less, the inward current decayed rapidly to a steady-state level during the light and returned to baseline shortly after the stimulus ceased (Fig. 1A, bottom 5 traces). As the stimulus intensity exceeded 9.5 log quanta/cm^2^/s, however, the post-stimulus inward current persisted for over 10 s, accompanied by a barrage of miniature EPSC events (Fig. 1A, top 4 traces). Similar results were observed in all 10 recorded dorsal DACs.

We also recorded 4 cells in the ventral retina. Figure 1B shows responses from a typical ventral DAC, demonstrating response sensitivity and dynamic characteristics similar to dorsal DACs. We then constructed peak-amplitude/intensity curves for dorsal and ventral DACs as described in the Methods (Fig. 1C). We noted several features in these curves. First, both dorsal and ventral DACs had a similar threshold intensity (6.5~7.5 log quanta/cm^2^/s) when presented with a white light stimulus. Second, dorsal and ventral DACs had almost the same peak response amplitude at low light intensities (7.5–8.5 log quanta/cm^2^/s). Third, the average peak amplitude of the dorsal DAC response tended to be larger than that of the ventral DAC response when the stimulus intensity exceeded 8.5 log quanta/cm^2^/s. Fourth, ventral DAC responses reached a relatively stable level between 10.5 and 11.5 log quanta/cm^2^/s, after which the responses slowly increased without reaching a plateau, even at 13.5 log quanta/cm^2^/s – the highest intensity provided by our system. Finally, dorsal DAC responses reached an initial peak at 11.5 log quanta/cm^2^/s; the response amplitude declined slightly at 12.5 log quanta/cm^2^/s and then increased again at 13.5 log quanta/cm^2^/s. The differences between the response curves for dorsal and ventral DACs suggest that DACs from these two regions may receive a distinct mix of inputs from rods, M-cones and melanopsin.

### Dorsal DAC responses mediated by rods, M-cones or melanopsin

**Figure 2:**
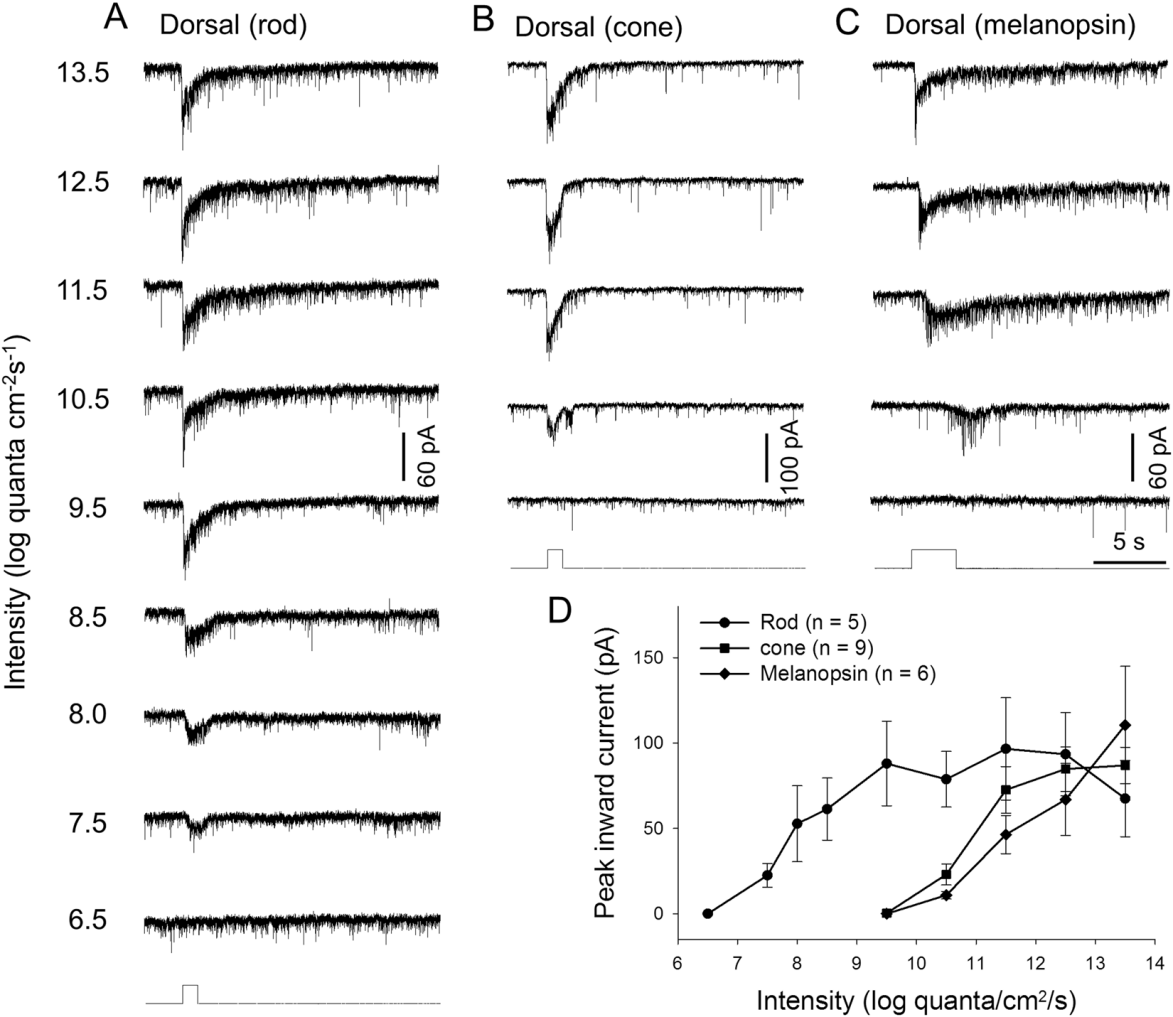
Relative contributions of rods, M-cones and melanopsin to dark-adapted dorsal DAC light responses. Light-evoked EPSCs of a dark-adapted dorsal DAC in a rod-function-only retina showing responses to a series of light stimuli ranging in intensity from 7.5 to 13.5 log quanta/cm^2^/s (**A**). M-cone-mediated responses of a DAC in a cone-function-only retina (**B**) demonstrate that its threshold intensity is three log units greater than that of rod-mediated responses. Melanopsin-based responses of a DAC (**C**) show the same threshold intensity as M-cone-mediated responses. Stimulation bar shows timing of light pulse (1-s duration, white light pulse). (**D**) Intensity-response profiles showing the average peak amplitudes of DAC light-evoked EPSCs as a function of the light intensity. Closed circles: rod-mediated responses; closed squares, M-cone-mediated responses; closed diamonds, melanopsin-based responses. Each data point shows the average and SE of multiple cells.

We then used genetic and pharmacological tools to isolate the contributions of rods, M-cones and melanopsin to dorsal DAC responses in dark-adapted retinae. Rod-driven responses were isolated by genetically silencing cone and melanopsin function in *TH*::RFP mice. In this mouse model, we found that DACs began to exhibit a light-induced EPSC at 7.5 log quanta/cm^2^/s (Fig. 2A), the same threshold intensity observed in WT dorsal DACs (Fig. 1A). The peak amplitude of the inward current reached a plateau around 9.5 log quanta/cm^2^/s (Fig. 2A,D). As the stimulus intensity exceeded 12.5 log quanta/cm^2^/s, however, the peak amplitude declined to some extent. These results show that in the dorsal retina, rods excite DACs at intensities ranging from 6.5 to 13.5 log quanta/cm^2^/s with a saturation intensity of 9.5 log quanta/cm^2^/s (Fig. 2D, n = 5). We also noted that after the peak amplitude reached a plateau at 9.5 log quanta/cm^2^/s, further increases in light intensity evoked responses that exhibited a pronounced post-stimulus persistence (Fig. 2A).

Next, cone function was isolated by genetically silencing rod and melanopsin function in *TH*::RFP mice^17^. We found that dorsal DACs began to respond to light at 10.5 log quanta/cm^2^/s, suggesting a threshold intensity between 9.5 and 10.5 log quanta/cm^2^/s (3 log units above the threshold of rod-mediated responses). The response reached a plateau at 12.5 log quanta/cm^2^/s (Fig. 2B,D, n = 9). At 13.5 log quanta/cm^2^/s, a post-stimulus inward current was observed, lasting approximately 2 s after stimulus termination (Fig. 2B).

Finally, we isolated melanopsin-based DAC responses in wild-type *TH*::RFP retinae using L-AP4 (50 µM), an agonist of group III metabotropic glutamate receptors that selectively blocks the retinal ON pathway^42^. We tested 10 DACs, of which 9 exhibited melanopsin-based responses. The threshold intensity of melanopsin-based EPSCs (9.5–10.5 log quanta/cm^2^/s, Fig. 2C,D) is similar to that of M-cone-mediated responses (Fig. 2B,D). The melanopsin-based response had a 2-s latency at 10.5 log quanta/cm^2^/s, with the latency gradually decreasing as the intensity increased (Fig. 2C). The rise time of the response at light onset and the response decay time during light stimulation gradually decreased as the light intensity increased (Fig. 2C). A prolonged post-stimulus component was observed in every response (Fig. 2C), as reported previously^18, 27, 30^. In addition, we noted that the peak amplitude of melanopsin-based DAC responses gradually increased as the stimulus intensity increased and did not reach a plateau, even at 13.5 log quanta/cm^2^/s (Fig. 2D).

### Ventral DAC responses mediated by rods, M-cones or melanopsin

**Figure 3:**
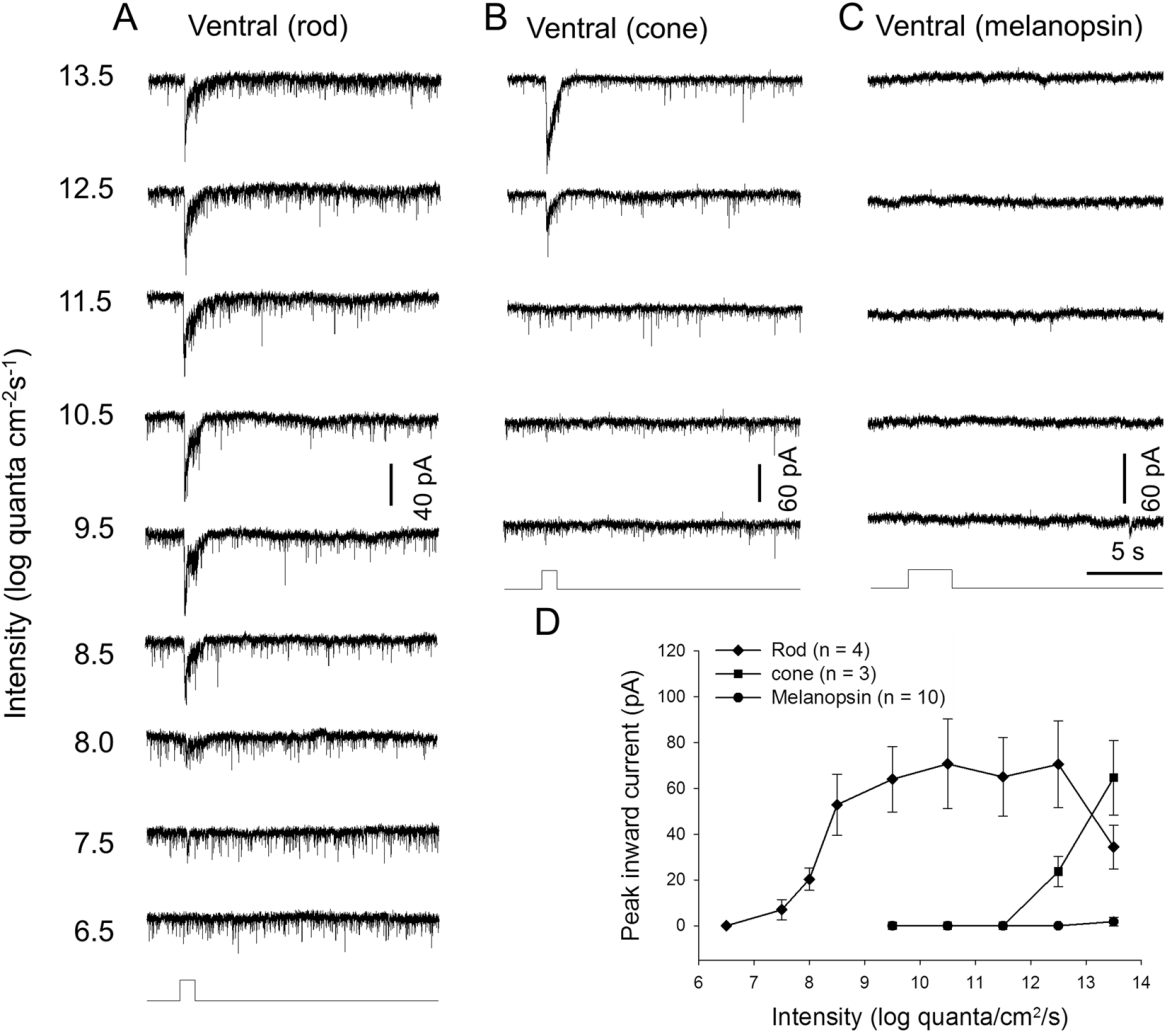
Relative contributions of rods, M-cones and melanopsin to dark-adapted ventral DAC light responses. Light-evoked EPSCs of a dark-adapted dorsal DAC in a rod-function-only retina showing the cell’s response to a series of light stimuli ranging in intensity from 7.5 to 13.5 log quanta/cm^2^/s (**A**). M-cone-mediated responses of a DAC in a cone-function-only retina (**B**) demonstrate that its threshold intensity is five log units greater than that of rod-mediated responses. Melanopsin-based responses of a DAC were undetectable in this cell (**C**). Stimulation bar shows timing of light pulse (1-s duration, white light pulse). (**D**) Intensity-response profiles showing the average peak amplitudes of DAC light-evoked EPSCs as a function of the light intensity. Closed circles: rod-mediated responses; closed squares, M-cone-mediated responses; closed diamonds, melanopsin-based responses. Each data point shows the average and SE of multiple cells.

We made recordings from ventral DACs in rod-function-only retinae and found that the sensitivity and dynamic characteristics of rod-driven responses in these cells were similar to those exhibited by dorsal DACs (Fig. 3A,D, n = 4). We also recorded from ventral DACs in cone-function-only retinae and found that the threshold intensity of M-cone-driven responses in ventral DACs was two log units higher than that observed in dorsal DACs (Fig. 3B,D, n = 3). Furthermore, we tested 11 ventral DACs for melanopsin-based responses in wild-type retinae in the presence of 50 µM L-AP4; however, only one cell showed a weak response at 13.5 log quanta/cm^2^/s. The remaining 10 cells showed no response at all intensities tested (Fig. 3C,D). To determine if longer stimulation could induce a response, we increased the stimulus duration from 3 s to 10 s. This prolonged stimulus failed to evoke any responses at 13.5 log quanta/cm^2^/s (data not shown). We also used epifluorescence light (~18 log quanta/cm^2^/s) but the epifluorescence stimulus failed to evoke any response from these cells (data not shown). These results suggest that the contribution of melanopsin to ventral DAC activity is very minor or nonexistent.

### Cx36-dependent and -independent rod-mediated DAC responses

**Figure 4:**
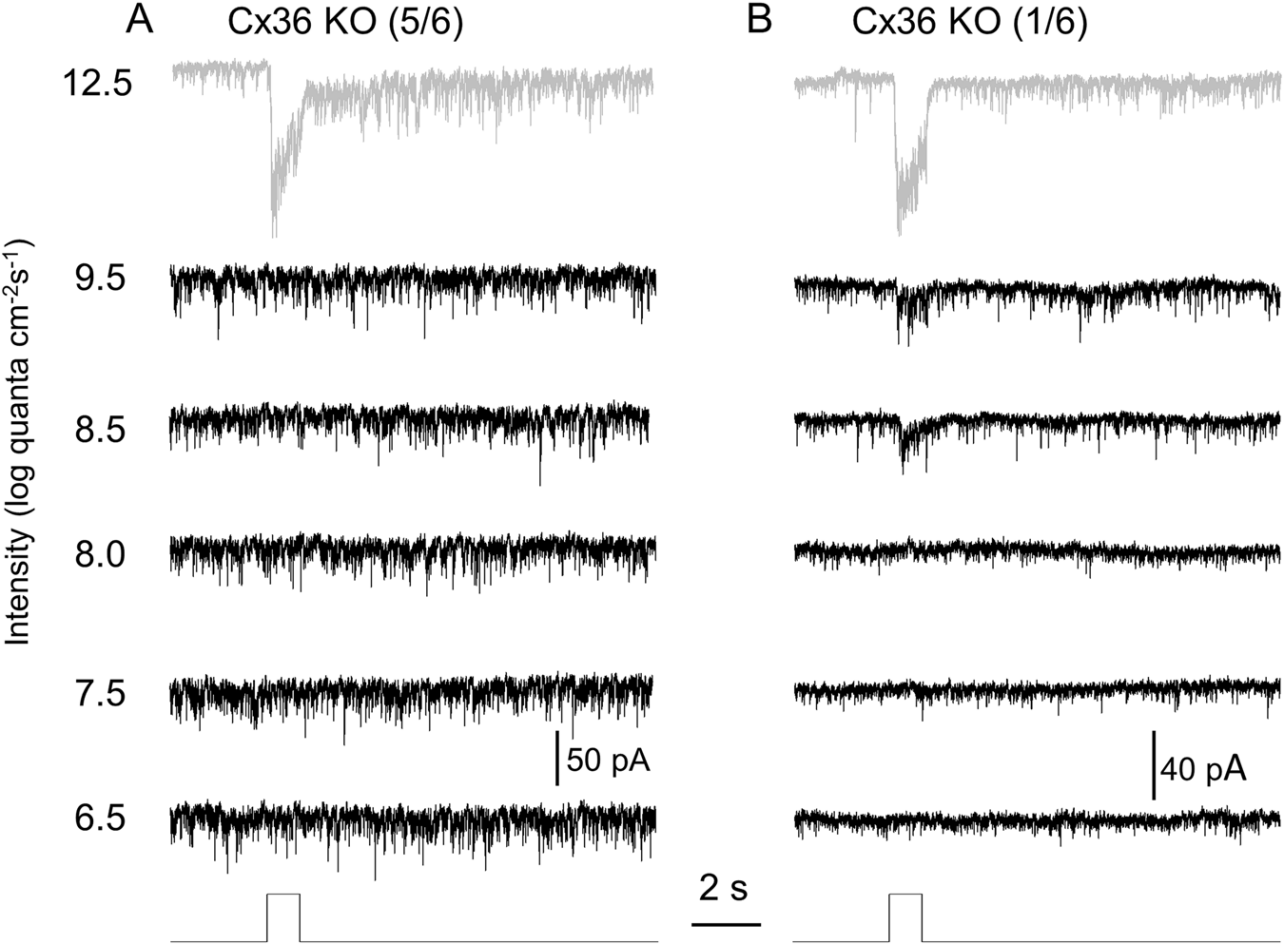
Rod-mediated DAC responses are eliminated or reduced in Cx36 KO mice. Six DACs were recorded in Cx36 KO retinae. (**A**) Five out of six DACs had no responses to a series of increasingly white light pulses (1-s duration) ranging from 6.5 to 9.5 log quanta/cm^2^/s (black traces). Top trace (gray) shows that the cell responded to a bright light pulse at 12.5 log quanta/cm^2^/s. (**B**) In contrast to the DAC shown in A, one DAC exhibited responses to light pulses at intensities of 8.5 and 9.5 log quanta/cm^2^/s (black traces). Top trace (gray) shows that the cell responded to a bright light pulse at 12.5 log quanta/cm^2^/s. Stimulation bar shows timing of light pulse.

We next determined the possible neural pathways that convey rod signals to DACs. It is not likely that rod signals reach third-order neurons directly through rod bipolar cells. Instead, they utilize at least three pathways to signal to cone bipolar cells, which then signal to third-order neurons^14^. The primary pathway consists of rods, rod bipolar cells and AII amacrine cells; the AII amacrine cells couple to cone bipolar cells via gap junctions. These gap junctions are exclusively mediated by Cx36^43^. The secondary pathway by which rods communicate with cone bipolar cells relies on rod signals passing directly to cones through rod-cone gap junctions. These gap junctions are also exclusively mediated by Cx36^44^. Finally, direct input from rods to cone bipolar cells forms the tertiary rod pathway^13, 45^. To determine whether Cx36-dependent and -independent rod pathways transmit rod signals to DACs, we examined the light-induced EPSCs of dorsal DACs in Cx36 KO retinae. We found that, in the majority of DACs, knocking out Cx36 eliminated DAC responses induced by light intensities ranging from 6.5 to 9.5 log quanta/cm^2^/s (Fig. 4A). As the stimulus intensity exceeded 9.5 log quanta/cm^2^/s, the peak amplitude of the DAC response gradually increased. The top trace of Fig. 4A is a response to a stimulus of 12.5 log units. Similar results were observed in 4 other dorsal DACs. Since rods are the only photosensitive cells producing responses in DACs at intensities ranging from 6.5 to 9.5 log quanta/cm^2^/s, our results suggest that the Cx36-dependent primary and secondary rod pathways convey rod signals to DACs. However, one exception is shown in Fig. 4B. This cell (from a Cx36 KO retina) did not respond to very dim light (6.5 and 7.5 log quanta/cm^2^/s) but exhibited responses at intensities of 8.5 and 9.5 log quanta/cm^2^/s. This result suggests that, in addition to Cx36-dependent pathways, the Cx36-independent tertiary rod pathway is likely to be involved in rod signal transmission to some DACs.

### Rod-mediated DAC responses to dim steady background or flashing light

**Figure 5:**
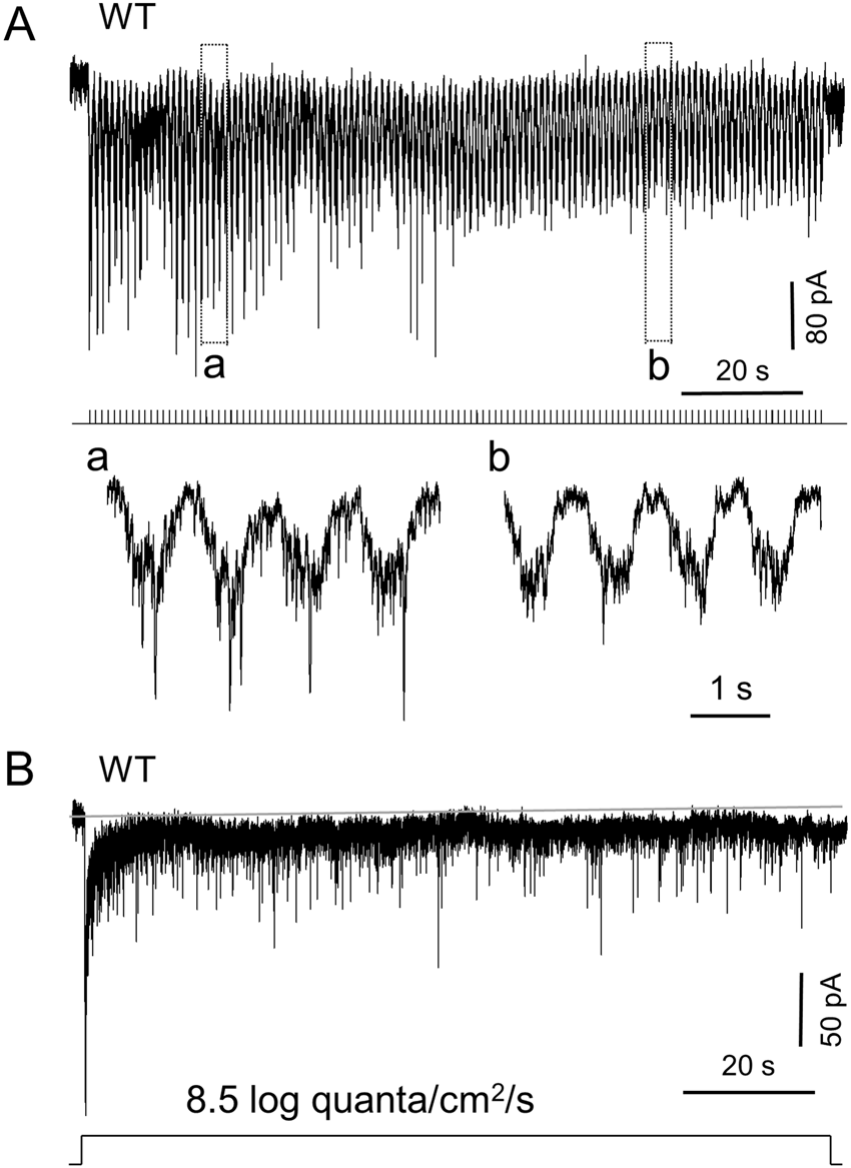
Rods contribute to DAC responses to dim steady background or flashing light. Experiments were conducted using wild-type mice. An intensity of 8.5 log quanta/cm^2^/s white light (which only activates rod photoreceptors) was used for stimuli. (**A**) A 500 ms-duration, 1 Hz flashing light was applied to the retina for 2 min. Traces in boxes a and b are time-expanded to show detailed response dynamics. The pattern of light stimuli is illustrated below the trace as a series of tick marks. (**B**) A rod-mediated DAC response was evoked using a 2 min steady background light stimulus. The sustained inward current evoked by the first light pulse remained constant throughout the 2 min stimulus. Solid gray line indicates the resting current baseline.

In the experiments above, we used a stimulus duration of 1 or 3 seconds to determine the relative contributions of rods, cones and melanopsin to light responses of dark-adapted DACs. However, dopamine has been implicated in processes of retinal light adaptation^46, 47^. The manner in which DACs respond to prolonged adapting light remains unclear. To address this question, we applied a steady background or low frequency (0.75~1 Hz) flashing light to the retina. Flashing background light elicits dopamine release in almost all species tested, whereas steady light only elicits dopamine release in some species^23, 26, 48–52^. Therefore, it is important to determine whether mouse DACs are able to respond to both stimuli. We first tested these adapting stimuli at low intensities to determine whether rods mediate light-adaptive DAC responses under scotopic conditions. Since cones and melanopsin are inactive under scotopic conditions, we used wild-type retinae to examine rod-mediated DAC responses. Because the threshold intensity of M-cone-mediated and melanopsin-based responses was >9.5 log quanta/cm^2^/s (Fig. 2D), we selected an experimental intensity of 8.5 log quanta/cm^2^/s to stimulate only rods. Figure 5A shows recordings from a dorsal DAC which responded well to a train of 1 Hz, 500 ms-duration pulses. The sustained inward current evoked by the first light pulse was maintained by subsequent pulses throughout the 2 min stimulation period and slowly returned to the baseline after the stimulus ceased. The repetitive responses evoked by the flashes were superimposed on a sustained inward current. The amplitudes of these repetitive responses gradually decreased during the first few pulses and then maintained a constant amplitude for the remainder of the stimulus (Fig. 5Aa,Ab). Similar results were observed in three other cells. When a steady background light with the same intensity was applied to a cell, the cell displayed a transient inward current which quickly decayed toward the baseline (Fig. 5B, n = 5). A sustained current of 5–10 pA persisted, accompanied by high-frequency EPSC events. This data shows that DACs respond to steady and flickering background light under scotopic conditions.

### Rod-, M-cone- and melanopsin-mediated DAC responses to intermediate intensity steady background or flashing light

When light intensities exceed the threshold intensity of cones, all three photosensitive cell classes are active. Since these photosensitive cell classes have distinct light sensitivities and response dynamics, we sought to determine their relative contributions to light-adaptive DAC responses under higher lighting conditions. In the next two sets of experiments, we tested two additional adapting lighting intensities that were above the cone threshold intensity: 11.5 log quanta/cm^2^/s (“intermediate”) and 13.5 log quanta/cm^2^/s (“bright”). All experiments were performed in dorsal DACs.

**Figure 6:**
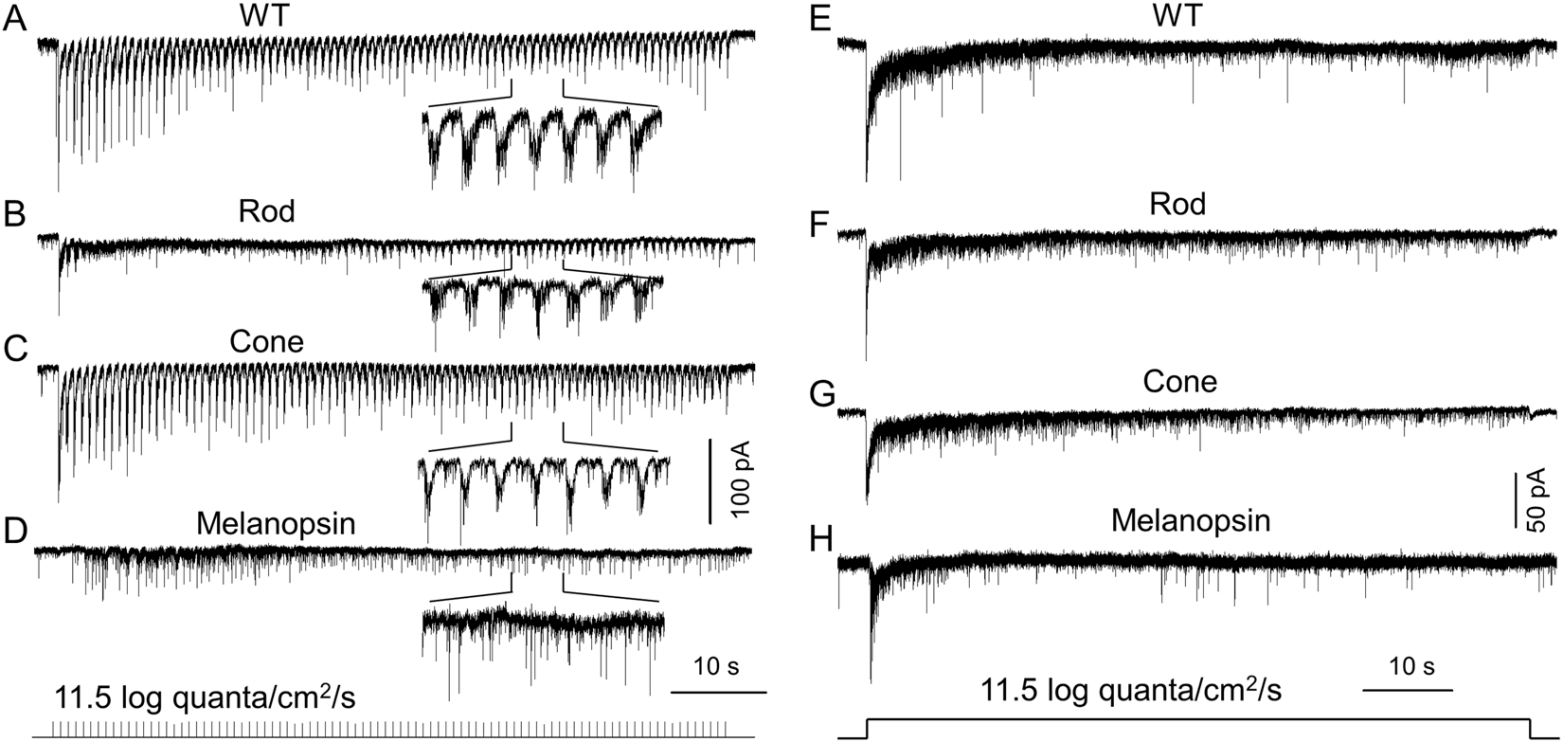
Rods, M-cones and melanopsin contribute to DAC responses to intermediate background light. (**A**–**D**) An 100 ms-duration, 0.75 Hz flashing light with an intensity of 11.5 log quanta/cm^2^/s was applied to the retina for 2 min. (**A**) Repetitive responses were obtained from a dorsal DAC in a wild-type retina. The indicated portion of the trace is time-expanded. (**B**) Rod-mediated responses of a DAC were recorded in the presence of TTX in a cone-function-knockout retina. The initial 3 pulses produced individual responses, after which the cell exhibited a steady-state inward current. The repetitive responses reappeared after approximately 40 s and continued until the stimulus was terminated. The indicated portion of trace is time-expanded. (**C**) Repetitive responses were obtained from a dorsal DAC in a cone-function-only retina. The indicated portion of trace is time-expanded. (**D**) The repetitive light pulses produced a barrage of melanopsin-based miniature EPSC events in a DAC. The cell was recorded in a wild-type retina in the presence of 50 µM L-AP4. The indicated portion of trace is time-expanded. A series of tick marks at the bottom of the figure indicate the pattern of light stimuli. (**E**–**H**) Steady background illumination with an intensity of 11.5 log quanta/cm^2^/s was applied to the retina for 2 min. A WT DAC had an initial inward current to the steady background light (**E**). The current slowly decayed toward the baseline. The same dynamic was observed in a rod-mediated DAC response (**F**), a M-cone-mediated response (**G**), and a melanopsin-based response (**H**). Stimulation bar shows timing of light pulse.

Using an intermediate stimulus intensity, we first tested a 0.75 Hz, 100 ms-duration flashing light on 4 dorsal DACs in wild-type mice. We found that the pulse response amplitudes were nearly constant for the first 10 to 12 stimuli, after which the response amplitude gradually decreased. The currents stabilized after about 40 s (Fig. 6A). Secondly, we measured the rod-mediated response to flashing light in 4 DACs from cone-function-knockout retinae in the presence of TTX, a blocker of signalling from ipRGCs to DACs^30^. We found that the first 3 stimuli produced individual responses, after which the cells exhibited only a steady-state inward current (Fig. 6B). This steady-state inward current lasted for approximately 40 s, after which the repetitive responses returned and continued until the stimulus was terminated (Fig. 6B). Thirdly, we examined M-cone-mediated DAC responses in cone-function-only retinae subjected to a flashing light. The responses we observed (Fig. 6C, n = 4) were very similar to those exhibited by wild-type retinae (Fig. 6A). Finally, melanopsin-based responses to flashing light were tested in wild-type retinae in the presence of 50µM L-AP4. The repeated light pulses produced a small sustained inward current accompanied by high-frequency EPSC events. However, responses to individual light pulses were absent (Fig. 6D, n = 7).

When a steady 11.5 log quanta/cm^2^/s background light was applied for 2 min, WT DACs displayed an initial inward current that slowly decayed, taking more than 30 seconds to stabilize (Fig. 6E, n = 4). In addition, the sustained response was accompanied by high levels of postsynaptic activity. These data suggest that under steady background illumination, WT DACs are constantly activated. Remarkably, we found that the dynamics of rod-mediated (Fig. 6F, n = 4), M-cone-mediated (Fig. 6G, n = 4), and melanopsin-based (Fig. 6H, n = 4) responses to steady background illumination were almost identical to those of WT DAC responses, except that rod-mediated responses had a quicker onset (Fig. 6F) and melanopsin-based responses had a slower onset (Fig. 6H). These results suggest that rods, M-cones and melanopsin contribute to the response of DACs under intermediate intensity adapting light stimulation.

### M-cone- and melanopsin-mediated responses to bright steady background or flashing light

In the next set of experiments, we increased the adapting light intensity to 13.5 log quanta/cm^2^/s. Here, we did not test the contribution of rods to the DAC response because the retina was not attached to the retinal pigment epithelium (RPE). Without the RPE, bleaching of rhodopsin by bright background light is irreversible, thereby preventing an accurate assessment of rod-driven responses.

**Figure 7:**
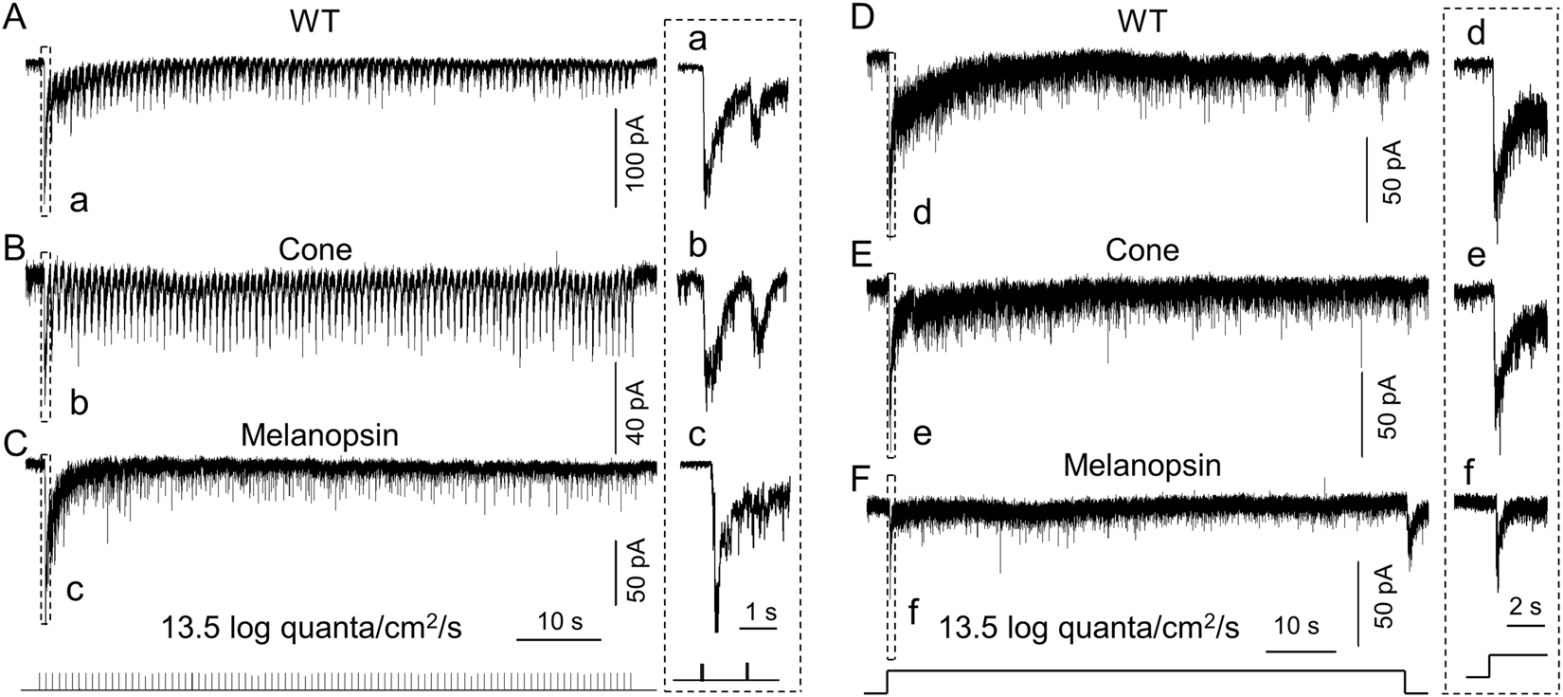
M-cones and melanopsin contribute to DAC responses to bright background light. (**A**–**C**) An 100 ms-duration, 0.75 Hz flashing light with an intensity of 13.5 log quanta/cm^2^/s was applied to the retina for 2 min. (**A**) Repetitive responses were obtained from a dorsal DAC in a wild-type retina. (**B**) Repetitive responses were obtained from a dorsal DAC in a cone-function-only retina. (**C**) The initial 3 pulses produced a melanopsin-based inward current followed by a steady-state desensitized current. The desensitized current was accompanied by a barrage of miniature EPSC events. Traces in box **a**, **b** and **c** are time-expanded and shown on the right. A series of tick marks at the bottom indicate the pattern of stimuli. (**D**–**F**) Steady background illumination with an intensity of 13.5 log quanta/cm^2^/s was applied to the retina for 2 min. A WT DAC had an initial inward current in response to steady background illumination (**D**). The current slowly decayed toward the baseline. The cone-mediated DAC response showed a peak current which was slowly desensitized toward the baseline (**E**). A melanopsin-based DAC response showed a peak current which was rapidly desensitized toward the baseline, followed by a steady-state current accompanied by EPSC events (**F**). The peak currents in box (d,e and f) are time-expanded and shown on the right. Stimulation bar shows timing of light pulse.

We found that WT DACs responded well to 0.75 Hz 100 ms-duration flashing light (Fig. 7A,a; n = 4). This stimulus produced a response somewhat similar to that recorded from DACs in cone-function-only retinae (Fig. 7B,b; n = 4). When we examined the contribution of melanopsin to DAC responses under these lighting conditions in WT retinae in the presence of 50 µM L-AP4, we found that the first light stimulus induced an inward current but that this current decayed toward the baseline within 10 seconds (Fig. 7C,c; n = 4). Further stimuli produced EPSC events which were superimposed on the desensitized current.

When a 13.5 log quanta/cm^2^/s steady background light was applied, WT DACs exhibited an inward current. Desensitization occurred over a 20-second period, resulting in a steady-state current of approximately 10 pA 1 min after light onset (Fig. 7D,d, n = 4). The dynamic characteristics of the DAC responses recorded in cone-function-only retinae were almost identical to those of WT DAC responses (Fig. 7E,e; n = 6). Furthermore, the melanopsin-based DAC response recorded from WT retinae in the presence of 50 µM L-AP4 quickly returned to the baseline, accompanied by EPSC events (Fig. 7F,f; n = 6). Collectively, these results suggest that cones and melanopsin contribute to DAC responses to bright adapting light stimuli.

## Discussion

**Figure 8:**
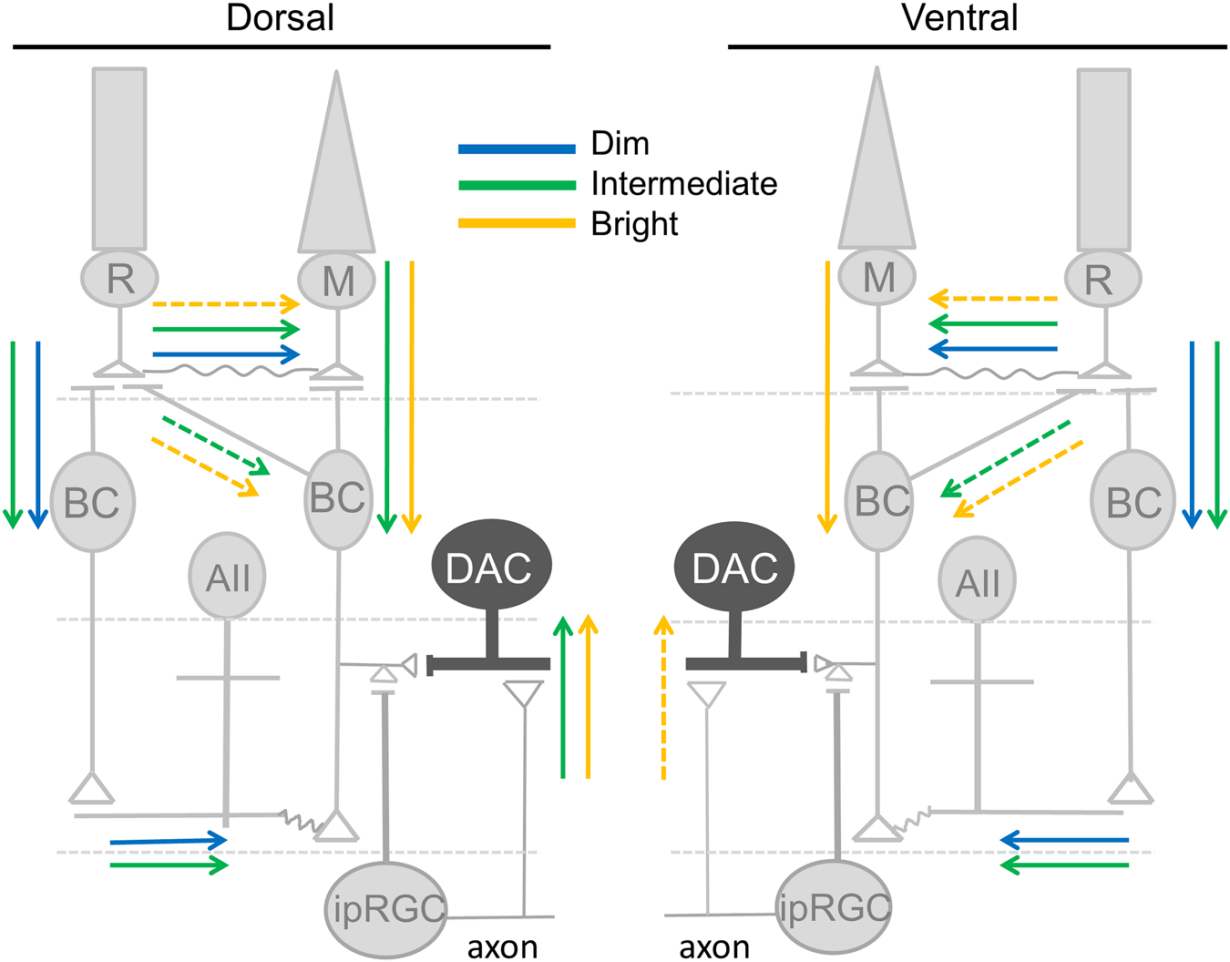
Proposed inputs from three distinct photoreceptor classes to DACs under different lighting conditions. The dorsal retinal network is illustrated on the left side; the ventral retinal network is depicted on the right side. R: rod; M: M-cone; BC: bipolar cell; AII: AII amacrine cell; DAC: dopaminergic amacrine cell; ipRGC: intrinsically photosensitive retinal ganglion cell. Under dim lighting conditions, DACs in the ventral and dorsal retina receive equal input from rods through the primary (rod ─ > rod BC ─ > AII amacrine ─ > cone BC) and secondary (rod ─ > cone) rod pathways (blue arrows) and possibly from the tertiary rod pathway (rod ─ > cone BC) (dashed blue line/arrow). Under intermediate lighting conditions, DACs in both the ventral and dorsal retina receive input from rods (green arrows). DACs in the dorsal retina also receive input from cones and melanopsin (green arrows). Under bright lighting conditions, DACs in the dorsal and ventral retina receive cone input (orange arrows). Additionally, DACs in the dorsal retina receive melanopsin input (orange arrow); melanopsin may contribute to DAC signalling in the ventral retina as well (dashed orange line/arrow). Under bright lighting conditions, rods may also excite DACs through the secondary and tertiary rod pathways (dashed orange line/arrow).

In the present study, we have characterized the relative contributions of rods, M-cones and ipRGCs to DAC light responses in the dark-adapted retina. As summarized in Fig. 8, we found that rods excite dorsal and ventral DACs equally across a wide range of light intensities via Cx36-dependent and -independent rod pathways. M-cones and melanopsin begin to stimulate dorsal DACs at the same threshold intensity, which is three log units higher than that of rods; however, their contributions to ventral DACs are much smaller than those to dorsal DACs. We further examined the contributions of rods, M-cones and melanopsin to light-adapted DAC responses and found that DAC light responses are mediated by rods under dim light conditions, rods/M-cones/melanopsin under intermediate conditions, and M-cones/melanopsin under bright light conditions. Our results provide insights into the cellular mechanisms responsible for dopamine release in different light adaptation states of the retina.

Dopamine has long been known to be involved in cone-mediated adaptation to bright light^46, 47^. However, dopamine also modulates the transmission of rod signals to post-receptoral neurons under dim light^53–56^. A logical consequence of this observation is that rods must trigger dopamine release from DACs under dim lighting conditions. Our results strongly support this hypothesis by demonstrating that rods produce excitatory currents in DACs in response to light over a wide range of stimulation intensities. We found the threshold intensity of rod-mediated DAC responses to be between 6.5 and 7.5 log quanta/cm^2^/s, three log units lower than the threshold of M-cone-mediated dorsal DAC responses. The saturation intensity of the rod-mediated DAC response was 9.5 log quanta/cm^2^/s, similar to the threshold intensity of M-cone-mediated responses observed in dorsal DACs. Therefore, light stimuli between 6.5~9.5 log quanta/cm^2^/s can be classified as scotopic stimuli. Within this range of stimulation intensities, rods are the only photoreceptors that excite DACs. Beyond the rod saturation intensity, rod-mediated responses in dorsal and ventral DACs maintained their maximum amplitude over a three-log-unit intensity range. Within this range, M-cones and melanopsin began to activate dorsal DACs (Fig. 2D), whereas the contribution of M-cones to ventral DACs was much smaller, and the contribution of melanopsin almost nonexistent (Fig. 3D). Therefore, at intensities ranging from 9.5 to 12.5 log quanta/cm^2^/s, the overall contribution of rods to DAC light responses may be greater than that of M-cones or melanopsin. However, the peak amplitude of rod-mediated DAC responses declined slightly at a stimulation intensity of 13.5 log quanta/cm^2^/s. This decline in response is likely a result of irreversible bleaching of rhodopsin due to the retina not being attached to the RPE.

Our data also show that rod-mediated DAC responses exhibit a post-stimulus component at higher stimulation intensities. A marked post-stimulus persistence has been previously reported in melanopsin-based DAC responses^27, 30^, which is validated in the present study. It can be surmised that the post-stimulus dorsal DAC responses observed in WT retinae are due not only to melanopsin signalling but also to stimulation from rods. Although we did not attempt to explore the mechanisms responsible for these rod-mediated post-stimulus DAC responses, it is highly likely that DACs inherit the response properties of rods, as mouse rods display a sagged response with a long plateau at saturating intensities^57^.

Within the scotopic range, we also examined rod-driven inhibitory postsynaptic currents (IPSCs) in WT DACs by holding the cell at 0 mV, which is the cationic reversal potential. We found that IPSCs had the same threshold intensity as EPSCs (data not shown). Since the IPSCs of DACs have been extensively studied by Newkirk *et al*.^18^, we did not investigate this subject further. However, we did note that Newkirk *et al*. found that the threshold intensity of EPSCs was 2 log units higher than that of IPSCs, which is inconsistent with our results. This difference could be due to the fact that we performed our recordings in dark-adapted retinae, while Newkirk *et al*. conducted experiments in the presence of background illumination that could eliminate EPSCs evoked by dimmer light.

There are at least three rod pathways in the mammalian retina (Fig. 8). The primary pathway (rod ─ > rod bipolar cell ─ > AII amacrine ─ > cone bipolar cell) carries the most sensitive rod signals while the secondary pathway (rod ─ > cone ─ > cone bipolar cell) transmits rod signals with slightly less sensitivity^58–63^. Deletion of Cx36 disrupts both the AII amacrine-ON bipolar cell gap junctions and the rod-cone gap junctions, resulting in the loss of signalling in the primary and secondary rod pathways^58, 62^. We found that genetic disruption of Cx36-mediated gap junctions eliminated rod-mediated responses in the majority of DACs (Fig. 4A), suggesting that the primary and secondary rod pathways are involved in transmitting rod signals to DACs. In addition, Cx36-independent rod-mediated responses to low stimulation intensities were observed in one DAC (Fig. 4B). Notably, the threshold sensitivity of the response was about two log units higher than that of the Cx36-dependent rod-mediated responses. These data indicate that rods could signal to some DACs directly through ON cone bipolar cells (the tertiary pathway) but only at relatively higher light intensities (Fig. 8)^13, 64, 65^. This conclusion is supported by physiological evidence of direct input from rods to ON cone bipolar cells^13^. This conclusion is also supported by the putative contacts between rods and type 7 ON cone bipolar cells observed in one study^45^ (however, these contacts were not observed in other studies^66, 67^). Moreover, we were unable to determine the neural pathways that convey rod signals to DACs at high lighting intensities, as they are mixed with cone and melanopsin signals in Cx36 KO retinae. However, we speculate that the secondary and tertiary rod pathways could be involved, as they operate at relatively high intensities (Fig. 8)^13, 20, 58, 64, 65^.

Our data demonstrate that rods contribute nearly equally to ventral and dorsal DAC light responses. This conclusion is supported by data indicating that the intensity-response relation of ventral DACs mirrors that of dorsal DACs in the rod-function-only retina (Figs 2D and 3D). In particular, ventral and dorsal DACs have the same threshold intensity and saturation intensity. The equal contribution of rods to dorsal and ventral DACs is also reflected in the intensity-response curves of WT ventral and dorsal DACs, as the two curves are almost identical within the scotopic intensity range.

In contrast to input from rods, the contribution of M-cones to ventral DACs is smaller than that to dorsal DACs, as evidenced by the fact that the threshold intensity of ventral DAC responses was two log units higher than that of dorsal DAC responses in the cone-function-only retina (Figs 2 and 3). This provides an explanation for the differing intensity-response relations of WT ventral and dorsal DACs at intensities ranging from 9.5 to 11.5 log quanta/cm^2^/s (Fig. 1C) ─ the contribution of M-cones to dorsal DACs makes their responses larger than those of ventral DACs.

The white light used consisted of three channels with emission peaks at 439, 515, and 582 nm, and thus excited M-opsin far more than S-opsin. M-opsin is expressed in a dorsal-ventral gradient^35–38, 68^; Wang *et al*. estimated that M-opsin expression drops from ~70% to <5% along the dorsal–ventral axis, with very low expression throughout the ventral retina^68^. The significantly lower amount of M-opsin in the ventral retina is likely to be the cause of the higher threshold observed for M-cone-mediated responses in ventral DACs. The difference in threshold sensitivity between ventral and dorsal DAC M-cone-mediated responses may also be a result of cone-mediated network differences (if any) between these two regions. Cones excite DACs directly via putative type 6 ON cone bipolar cells or indirectly through ipRGCs^17, 69^. It is not known if cone bipolar cells, in particular types 6 and 7^45, 69^, network differently with M-cones in the ventral retina than in the dorsal retina. However, our data show that input from ipRGCs to DACs is more pronounced in the dorsal retina than in the ventral retina (see discussion below). As a result, cones may excite DACs via ipRGCs to a greater extent in the dorsal retina than in the ventral retina.

The majority of dorsal DACs exhibited melanopsin-based responses with a threshold intensity between 9.5 and 10.5 log quanta/cm^2^/s. This threshold intensity range appears to be the same as that of M1 ipRGCs, which we have studied previously^41^. In addition, we found that the threshold intensity of the melanopsin-based response is near that of the M-cone-mediated response in dorsal DACs. This finding seems to contradict previous studies, which concluded that the sensitivity of melanopsin was about four log units higher than that of cones^70, 71^. This difference may be due to the use of a multiphoton laser (rather than epifluorescence) to image fluorescently labeled cells^41, 71^. Multiphoton imaging minimizes photobleaching, which may have allowed us to observe a higher sensitivity of melanopsin due to the dark-adapted state of the retina^41^.

Unlike the M-cone-based response, the amplitude of the melanopsin-based response increased linearly with the increase in light intensity, suggesting that the response saturation intensity is far from the maximum intensity of 13.5 log quanta/cm^2^/s used for the present study. This linear relationship exactly matches the intensity-response relations for melanopsin-based M1 ipRGC responses reported previously^41^, further supporting the hypothesis that DACs receive input from this subtype of ipRGCs^30^. The contribution of melanopsin to dorsal DACs could explain the increase in WT dorsal DAC response amplitude at 13.5 log quanta/cm^2^/s (Fig. 1C). In contrast, we rarely observed melanopsin-based signals in ventral DACs. The differential contribution of melanopsin to ventral and dorsal DACs is consistent with previous work showing that melanopsin-based c-fos expression predominates in the dorsal region of the rodless/coneless retina^31^. Although it has been reported that there are more ipRGCs in the dorsal retina than in the ventral retina^40^, there are still a significant number of ipRGCs in the ventral retina which could drive DACs. Therefore, the uneven distribution of melanopsin between the ventral and dorsal retina is not enough to account for the lack of significant melanopsin-based responses in ventral DACs. Instead, we speculate that ipRGC axon collaterals, which have previously been reported to be presynaptic to DACs^30, 72^, project mainly to the dorsal retina.

Direct measurements of dopamine release have demonstrated that dopamine release is elevated under dim background light^4, 19^. Electrophysiological evidence has also suggested that AII amacrine cells are uncoupled by light at the high end of the scotopic range, possibly through the release of dopamine^73, 74^. Our data support these findings, as we have demonstrated that under dim background illumination, both steady and low-frequency flashing background light can signal through rods to excite DACs. It appears that flashing light is more efficient than steady background light in inducing DAC responses. This is likely due to the fact that DACs exhibit rapid desensitization under steady background lighting (Fig. 5B).

Under intermediate intensity lighting conditions, rods, M-cones, and melanopsin are all involved in driving DACs in the dorsal retina. However, for a flashing background light stimulus, the contribution of M-cones was more significant than that of rods or melanopsin. The rod-mediated response was saturated after the first few flashes and then reappeared after a period of time. Since rods are saturated by this stimulus, the reappearance of the response is likely mediated by retinal network adaptation. The rod-mediated response could drive dopamine release to enable cone-like spatial visual function, as previously reported^19, 22, 75^. In contrast, the contributions of rods, M-cones and melanopsin are quite similar under steady background light. If we add up the responses mediated by rods, M-cones, and melanopsin (Fig. 6B–D), the sum total appears to be much greater than the response observed in WT DACs (Fig. 6A). This clearly demonstrates that synaptic inputs to dorsal DACs from rods, M-cones and ipRGCs are integrated in a nonlinear manner. Defining the possible sites and mechanisms of the integration is beyond the scope of the present study, but possible sites could include rod-cone couplings, photoreceptor-bipolar cell synapses, bipolar cell-DAC synapses, and/or ipRGC-DAC synapses. Presynaptic Ca^2+^ channels likely play a critical role in narrowing the synapse operating range because of their narrow activation voltage window^76, 77^. Postsynaptic glutamate receptors and electrical synapses may further enhance nonlinear presynaptic integration by way of receptor desensitization and voltage-dependent inactivation, respectively^28, 78–80^.

Under bright light conditions, both steady and flickering background light produced remarkable cone-mediated responses, suggesting that cones are a significant contributor to light-adapted DAC responses in the WT retina. This supports our previous work showing that cones are able to stimulate dopamine release at photopic stimulus intensities without the help of other photoreceptors^17^, possibly mediating light adaptation and contrast sensitivity in the visual system^75^. In addition, at the highest intensity we tested, the contribution of melanopsin appears to be smaller than the contribution of cones for both steady and flickering background light stimuli. Since we do not know the exact saturation intensity of the melanopsin-based response, melanopsin may contribute to an even greater extent at higher stimulation intensities. Although the role of melanopsin in visual processing is still under investigation, under bright light conditions, melanopsin could modulate signal transmission in the rod and cone pathways through dopamine signalling^30, 81, 82^. Finally, we were not able to determine the contribution of rods to DAC responses to bright background light in the *in vitro* mouse retina. However, we noted an unpublished study showing that a high-threshold (>1000 lux) rod input drives retinal dopamine release in response to light *in vivo* (Victor *et al*., 2017 ARVO Annual Meeting Abstract). Therefore, it appears that the contributions of rods to DAC activity and retinal dopamine release are more complicated than previously thought. This issue deserves a thorough investigation in the future.

## Materials and Methods

### Animals

Adult male and female mice (2–4 months old) were used for all experiments. The mice were housed in the Oakland University animal facility on a 12:12-h light-dark cycle, with lights on at 07:30 h. Mice were delivered to the University of Michigan, where experiments were conducted. All procedures conformed to NIH guidelines for laboratory animals and were approved by the Institutional Animal Care and Use Committees at the Oakland University and the University of Michigan.

A *TH*::RFP mouse line with a C57BL/6J background was obtained from Vanderbilt University^83^. The mice were crossed with a triple-knockout mouse line (BL6/129) in which the cone photoreceptor-specific cyclic nucleotide channel *Cnga3*, rod-specific G protein transducin α-subunit *Gnat1*, and photopigment melanopsin *Opn4* were deleted^84^. From multiple crossings, we bred *Cnga3* ^−/−^ *TH*::RFP, *Cnga3* ^−/−^ *Opn4* ^−/−^ *TH::*RFP, *Gnat1* ^−/−^ *Opn4* ^−/−^ *TH::*RFP, and *Gnat1* ^+/+^ *Cnga3* ^+/+^ *Opn4* ^+/+^ *TH::*RFP mouse lines for the present study. *Gnat1* ^+/+^ *Cnga3* ^+/+^ *Opn4* ^+/+^ *TH::*RFP mice (BL6/129 + C57BL/6 J) were further crossed with Cx36 KO mice (C57BL/6 J)^85^ to produce Cx36 KO *TH*::RFP mice. This new mouse line thus had a mixed BL6/126 and C57BL/6 J background.

### Electrophysiological recording

Whole-cell recording and two-photon imaging procedures were identical to those described previously^41^, except that recordings were made from RFP-labeled somata in the inner nuclear layer, and that all voltage-clamp recordings used a Cs^+^-based intracellular solution containing (in mM): 120 Cs-methane sulfonate, 5 EGTA, 10 HEPES, 5 CsCl, 5 NaCl, 0.5 CaCl_2_, 4 Na-ATP, 0.3 Na-GTP, and 5 lidocaine n-ethyl-chloride (QX-314), pH adjusted to 7.3 with CsOH. QX-314 was used to improve the space clamp quality of the voltage-clamp as well as to highlight the light-induced inward current of the cells by blocking intrinsic Na^+^ channel-mediated action potentials.

### Light stimulus

The light source was a miniature OLED monitor (SVGA Rev. 2; eMagin, Bellevue, WA, USA), which had three channels with emission peaks at 439, 515 and 582 nm. This monitor was attached to a filter holder mounted on the camera port of the microscope. The light intensity was adjusted by means of neutral density filters inserted into the holder. All stimuli were full-field (~3 mm diameter) white light generated by activating all three colour channels. The unattenuated white light was calibrated to be equivalent to 13.5 log quanta/cm^2^/s of 515 nm light. All photon fluxes in the text are expressed as 515 nm light equivalent. Stimuli were programmed in Matlab using the Psychophysics Toolbox and delivered to the retina through the objective lens. Due to its lack of ultraviolet emission, this light source likely stimulated S-cone opsin far less than M-cone opsin.

### Data analyses

Data were analysed offline using the Clampfit 10.4 (Molecular Devices, Sunnyvale, CA) and SigmaPlot 12.0 (Systat Software, Germany) software packages. The peak amplitude of the EPSC from each DAC was measured and the peak currents from different cells at the same light intensity were then averaged. The mean peak currents were plotted against light stimulus intensity to construct a peak-intensity/response curve. Data are presented as mean ± SEM.

### Data availability

All data generated or analysed during this study are included in this published article.
